# Medical negligence liability under the consumer protection act: A review of judicial perspective

**DOI:** 10.4103/0970-1591.56205

**Published:** 2009

**Authors:** S. V. Joga Rao

**Affiliations:** Formerly Additional Professor of Law, National Law School of India University, Bangalore, India

**Keywords:** Cconsumer protection act, negligence, reasonable care

## Abstract

It is important to know what constitutes medical negligence. A doctor owes certain duties to the patient who consults him for illness. A deficiency in this duty results in negligence. A basic knowledge of how medical negligence is adjudicated in the various judicial courts of India will help a doctor to practice his profession without undue worry about facing litigation for alleged medical negligence.

## INTRODUCTION

Lately, Indian society is experiencing a growing awareness regarding patient's rights. This trend is clearly discernible from the recent spurt in litigation concerning medical professional or establishment liability, claiming redressal for the suffering caused due to medical negligence, vitiated consent, and breach of confidentiality arising out of the doctor-patient relationship. The patient-centered initiative of rights protection is required to be appreciated in the economic context of the rapid decline of State spending and massive private investment in the sphere of the health care system and the Indian Supreme Court's painstaking efforts to Constitutionalize a right to health as a fundamental right. As of now, the adjudicating process with regard to medical professional liability, be it in a consumer forum or a regular civil or criminal court, considers common law principles relating to negligence, vitiated consent, and breach of confidentiality. However, it is equally essential to note that the protection of patient's right shall not be at the cost of professional integrity and autonomy. There is definitely a need for striking a delicate balance. Otherwise, the consequences would be inexplicable.

In the context of obtaining processes, there is a deserving need for a two-pronged approach. On one hand, the desirable direction points towards identification of minimum reasonable standards in light of the social, economical, and cultural context that would facilitate the adjudicators to decide issues of professional liability on an objective basis. On the other hand, such identification enables the medical professionals to internalize such standards in their day-to-day discharge of professional duties, which would hopefully prevent to a large extent the scenario of protection of patient's rights in a litigative atmosphere. In the long run, the present adversarial placement of doctor and the patient would undergo a transformation to the advantage of the patient, doctor, and society at large.

## WHAT A MEDICAL DOCTOR SHOULD KNOW ABOUT COPRA?

### Who can file a complaint?

A consumer or any recognized consumer association, i.e., voluntary consumer association registered under the Companies Act, 1956 or any other law for the time being in force, whether the consumer is a member of such association or not, or the central or state government.

### Who is a consumer?

A consumer is a person who hires or avails of any services for a consideration that has been paid or promised or partly paid and partly promised or under any system of deferred payment and includes any beneficiary of such services other than the person hires or avails of the services for consideration paid or promised, or under any system of deferred payment, when such services are availed of with the approval of the first mentioned person. This definition is wide enough to include a patient who merely promises to pay.

### What is a complaint?

A complaint is an allegation in writing made by a Complainant, i.e., a consumer that he or she has suffered loss or damage as a result of any deficiency of service.

### What is deficiency of service?

Deficiency of service means any fault, imperfection, shortcoming, or inadequacy in the quality, nature, or manner of performance that is required to be maintained by or under any law for the time being in force or has been undertaken to be performed by a person in pursuance of a contract or otherwise in relation to any service.

### Where is a complaint filed?

A complaint can be filed in 1) the District Forum if the value of services and compensation claimed is less than 20 lakh rupees, 2) before the State Commission, if the value of the goods or services and the compensation claimed does not exceed more than 1 crore rupees, or 3) in the National Commission, if the value of the goods or services and the compensation exceeds more than 1 crore rupees.

### What is the cost involved in filing a complaint?

There is a minimal fee for filing a complaint before the district consumer redressal forums.

### Is there any provision for appeal?

An appeal against the decision of the District Forum can be filed before the State Commission. An appeal will then go from the State Commission to the National Commission and from the National Commission to the Supreme Court. The time limit within which the appeal should be filed is 30 days from the date of the decision in all cases.

### What are the powers of the consumer redressal forums?

The forums have a variety of powers. They are 1) the summoning and enforcing of the attendance of any defendant or witness and examining the witness under oath, 2) the discovery and production of any document or other material object producible as evidence, 3) the reception of evidence on affidavits, 4) the summoning of any expert evidence or testimony, 5) the requisitioning of the report of the concerned analysis or test from the appropriate laboratory or from any other relevant source, 6) issuing of any commission for the examination of any witness, and 7) any other matter which may be prescribed.

### How does adjudication of liability take place?

The process before the competent forum will be set in motion in the following manner. When the Complainant files a written complaint, the forum, after admitting the complaint, sends a written notice to the opposite party asking for a written version to be submitted within 30 days. Thereafter, subsequent to proper scrutiny, the forum would ask for either filing of an affidavit or production of evidence in the form of interrogatories, expert evidence, medical literature, and judicial decisions.

## MEDICAL NEGLIGENCE - DEFINITIONAL ASPECTS

Negligence is simply the failure to exercise due care. The three ingredients of negligence are as follows:

1. The defendant owes a duty of care to the plaintiff.
2. The defendant has breached this duty of care.
3. The plaintiff has suffered an injury due to this breach.

Medical negligence is no different. It is only that in a medical negligence case, most often, the doctor is the defendant.

### When does a duty arise?

It is well known that a doctor owes a duty of care to his patient. This duty can either be a contractual duty or a duty arising out of tort law. In some cases, however, though a doctor-patient relationship is not established, the courts have imposed a duty upon the doctor. In the words of the Supreme Court “every doctor, at the governmental hospital or elsewhere, has a professional obligation to extend his services with due expertise for protecting life” (Parmanand Kataria *vs*. Union of India[1]). These cases are however, clearly restricted to situations where there is danger to the life of the person. Impliedly, therefore, in other circumstances the doctor does not owe a duty.

### What is the duty owed?

The duty owed by a doctor towards his patient, in the words of the Supreme Court is to “bring to his task a reasonable degree of skill and knowledge” and to exercise “a reasonable degree of care” (Laxman *vs*. Trimback[2]). The doctor, in other words, does not have to adhere to the highest or sink to the lowest degree of care and competence in the light of the circumstance. A doctor, therefore, does not have to ensure that every patient who comes to him is cured. He has to only ensure that he confers a reasonable degree of care and competence.

### Reasonable degree of care

Reasonable degree of care and skill means that the degree of care and competence that an “ordinary competent member of the profession who professes to have those skills would exercise in the circumstance in question.” At this stage, it may be necessary to note the distinction between the standard of care and the degree of care. The standard of care is a constant and remains the same in all cases. It is the requirement that the conduct of the doctor be reasonable and need not necessarily conform to the highest degree of care or the lowest degree of care possible. The degree of care is a variable and depends on the circumstance. It is used to refer to what actually amounts to reasonableness in a given situation.

Thus, though the same standard of care is expected from a generalist and a specialist, the degree of care would be different. In other words, both are expected to take reasonable care but what amounts to reasonable care with regard to the specialist differs from what amount of reasonable care is standard for the generalist. In fact, the law expects the specialist to exercise the ordinary skill of this speciality and not of any ordinary doctor. Though the courts have accepted the need to impose a higher degree of duty on a specialist, they have refused to lower it in the case of a novice.

Another question that arises is with regard to the knowledge that is expected from a doctor. Should it include the latest developments in the field, hence require constant updating or is it enough to follow what has been traditionally followed? It has been recognized by the courts that what amounts to reasonableness changes with time. The standard, as stated clearly herein before requires that the doctor possess reasonable knowledge. Hence, we can conclude that a doctor has to constantly update his knowledge to meet the standard expected of him. Furthermore, since only reasonable knowledge is required, it may not be necessary for him to be aware of all the developments that have taken place.

We have, until now, examined the duty of a doctor in so far as treating a patient is concerned or in diagnosing the ailment. Doctors are, however, imposed with a duty to take the consent of a person/patient before performing acts like surgical operations and in some cases treatment as well. To summarize, any act that requires contact with the patient has to be consented by the patient. A duty of care is imposed on the doctors in taking the patient's consent. Naturally, a question arises as to what is this duty of care. As per the judicial pronouncements, this duty is to disclose all such information as would be relevant or necessary for the patient to make a decision. Therefore, the duty does not extend to disclosing all possible information in this regard. Furthermore, this duty does not extend to warning a patient of all the normal attendant risks of an operation. The standard of care required of a doctor while obtaining consent is again that of a reasonable doctor, as in other cases.

### When does the liability arise?

The liability of a doctor arises not when the patient has suffered any injury, but when the injury has resulted due to the conduct of the doctor, which has fallen below that of reasonable care. In other words, the doctor is not liable for every injury suffered by a patient. He is liable for only those that are a consequence of a breach of his duty. Hence, once the existence of a duty has been established, the plaintiff must still prove the breach of duty and the causation. In case there is no breach or the breach did not cause the damage, the doctor will not be liable. In order to show the breach of duty, the burden on the plaintiff would be to first show what is considered as reasonable under those circumstances and then that the conduct of the doctor was below this degree. It must be noted that it is not sufficient to prove a breach, to merely show that there exists a body of opinion which goes against the practice/conduct of the doctor.

With regard to causation, the court has held that it must be shown that of all the possible reasons for the injury, the breach of duty of the doctor was the most probable cause. It is not sufficient to show that the breach of duty is merely one of the probable causes. Hence, if the possible causes of an injury are the negligence of a third party, an accident, or a breach of duty care of the doctor, then it must be established that the breach of duty of care of the doctor was the most probable cause of the injury to discharge the burden of proof on the plaintiff.

Normally, the liability arises only when the plaintiff is able to discharge the burden on him of proving negligence. However, in some cases like a swab left over the abdomen of a patient or the leg amputated instead of being put in a cast to treat the fracture, the principle of ‘res ipsa loquitur’ (meaning thereby ‘the thing speaks for itself’) might come into play. The following are the necessary conditions of this principle.

1. Complete control rests with the doctor.
2. It is the general experience of mankind that the accident in question does not happen without negligence. This principle is often misunderstood as a rule of evidence, which it is not. It is a principle in the law of torts. When this principle is applied, the burden is on the doctor/defendant to explain how the incident could have occurred without negligence. In the absence of any such explanation, liability of the doctor arises.

Normally, a doctor is held liable for only his acts (other than cases of vicarious liability). However, in some cases, a doctor can be held liable for the acts of another person which injures the patient. The need for such a liability may arise when the person committing the act may not owe a duty of care at all to the patient or that in committing the act he has not breached any duty. A typical example of a case where such a situation may arise is in the case of a surgery. If a junior doctor is involved as part of the team, then his duty, as far as the exercise of the specialist skill is concerned, is to seek the advice or help of a senior doctor. He will have discharged his duty once he does this and will not be liable even if he actually commits the act which causes the injury. In such a case, it is the duty of the senior doctor to have advised him properly. If he did not do so, then he would be the one responsible for the injury caused to the patient, though he did not commit the act.

### When there is no liability

A doctor is not necessarily liable in all cases where a patient has suffered an injury. This may either be due to the fact that he has a valid defense or that he has not breached the duty of care. Error of judgment can either be a mere error of judgment or error of judgment due to negligence. Only in the case of the former, it has been recognized by the courts as not being a breach of the duty of care. It can be described as the recognition in law of the human fallibility in all spheres of life. A mere error of judgment occurs when a doctor makes a decision that turns out to be wrong. It is situation in which only in retrospect can we say there was an error. At the time when the decision was made, it did not seem wrong. If, however, due consideration of all the factors was not taken, then it would amount to an error of judgment due to negligence.

## JUDICIAL INTERPRETATION OF MEDICAL NEGLIGENCE LIABILITY

By and large the following legal issues have been addressed and responded to by different forums and Courts in India.

### Charge of Medical Negligence against Professional Doctors

From the time of Lord Denning until now it has been held in several judgments that a charge of professional negligence against the medical professional stood on a different footing from a charge of negligence against the driver of a motor car. The burden of proof is correspondingly greater on the person who alleges negligence against a doctor. It is a known fact that with the best skill in the world, things sometimes went wrong in medical treatment or surgical operation. A doctor was not to be held negligent simply because something went wrong. The National Commission as well as the Apex Court in catena of decisions has held that the doctor is not liable for negligence because of someone else of better skill or knowledge would have prescribed a different treatment or operated in a different way. He is not guilty of negligence if he has acted in accordance with the practice accepted as proper by a reasonable body of medical professionals. The Hon'ble Supreme Court in the case of Dr. Laxman Balkrishna *vs*. Dr. Trimbak, AIR 1969 SC 128, has held the above view that is still considered to be a landmark judgment for deciding a case of negligence. In the case of Indian Medical Association *vs*. Santha, the Apex Court has decided that the skill of a medical practitioner differs from doctor to doctor and it is incumbent upon the Complainant to prove that a doctor was negligent in the line of treatment that resulted in the life of the patient. Therefore, a Judge can find a doctor guilty only when it is proved that he has fallen short of the standard of reasonable medical care. The principle of Res-Ipsa-Loquitur has not been generally followed by the Consumer Courts in India including the National Commission or even by the Apex Court in deciding the case under this Act. In catena of decisions, it has been held that it is for the Complainant to prove the negligence or deficiency in service by adducing expert evidence or opinion and this fact is to be proved beyond all reasonable doubts. Mere allegation of negligence will be of no help to the Complainant.[3]

### What Constitutes Medical Negligence?

Failure of an operation and side effects are not negligence. The term negligence is defined as the absence or lack of care that a reasonable person should have taken in the circumstances of the case. In the allegation of negligence in a case of wrist drop, the following observations were made. Nothing has been mentioned in the complaint or in the grounds of appeal about the type of care desired from the doctor in which he failed. It is not said anywhere what type of negligence was done during the course of the operation. Nerves may be cut down at the time of operation and mere cutting of a nerve does not amount to negligence. It is not said that it has been deliberately done. To the contrary it is also not said that the nerves were cut in the operation and it was not cut at the time of the accident. No expert evidence whatsoever has been produced. Only the report of the Chief Medical Officer of Haridwar has been produced wherein it said that the patient is a case of post-traumatic wrist drop. It is not said that it is due to any operation or the negligence of the doctor. The mere allegation will not make out a case of negligence, unless it is proved by reliable evidence and is supported by expert evidence. It is true that the operation has been performed. It is also true that the Complainant has many expenses but unless the negligence of the doctor is proved, she is not entitled to any compensation.[4]

### What is the Standard of Care?

It is now a settled principle of law that a medical practitioner will bring to his task a reasonable degree of skill and knowledge and must exercise a reasonable degree of care. Neither the very highest nor the very lowest degree of care and competence judged in the light of circumstances in each case is what the law requires. Judged from this yardstick, post-operative infection or shortening of the leg was not due to any negligence or deficiency in service on the part of the opposite party Appellant. Deficiency in service thus cannot be fastened on the opposite party.[5]

In a case that led to visual impairment as a side effect, the following observations were made. The literature with regard to lariago clearly mentioned that the side effect of this medicine if taken for a longer duration can effect eyesight but this is not a fact in this case. Besides, there is no expert evidence on record to show that use of this medicine caused damage to the patient's eyesight. Even for argument's sake, if it is accepted that this medicine caused damage to the patient's eyesight, if the Respondent-doctor is one who has advised his patient to use this medicine after an examination in which he found the patient to be suffering from malaria, in that case as well the doctor-Respondent cannot be held guilty of negligence or deficient in his service. However, as stated above in this case the medicine has been used by the patient in low doses for a few days and there is no expert evidence to show that the use of medicine has affected his eyesight. Therefore, the Complainant-Appellant has failed to prove that the Respondent was negligent and deficient in his duty as a doctor.[6]

### Proof of Medical Negligence

It has been held in different judgments by the National Commission and by the Hon'ble Supreme Court that a charge of professional negligence against a doctor stood on a different footing from a charge of negligence against a driver of a vehicle. The burden of proof is correspondingly greater on the person who alleges negligence against a doctor. It is a known fact that even with a doctor with the best skills, things sometimes go wrong during medical treatment or in a surgery. A doctor is not to be held negligent simply because something went wrong. It is an admitted fact that the Complainant's eyesight was not restored after the operation was conducted by the Appellant but on this ground alone a doctor can not be held negligent because even after adopting all necessary precautions and care the result of the operation may not be satisfactory since it depends on various other factors. The contention of the Appellant was that the patient was suffering from diabetes and blood pressure and in many such cases eyesight is not restored after the operation however carefully it is done. In this case, there is nothing on record to show that something went wrong due to an act of the Appellant-doctor. There is no evidence to come to the conclusion that the Appellant fell below the standard of a reasonably competent practitioner in their field, so much so that their conduct might be deserving of censure. The Appellant cannot be liable for negligence because someone else of better skill or knowledge would have prescribed a different method of operation in different way. The evidence suggests that the Appellant has performed the operation and acted in accordance with the practice regularly accepted and adopted by him in this hospital and several patients are regularly treated for their eye problems. The Hon'ble Supreme Court in the case of Dr. Laxman Balkrishna *vs*. Dr. Triambak, AIR 1969 Supreme Court page 128 has held the above view and this view has been further confirmed in the case of the Indian Medical Association *vs*. Santha. The Apex Court and the National Commission has held that the skill of a medical practitioner differs from doctor to doctor and it is an incumbent upon the Complainant to prove that the Appellant was negligent in the line of treatment that resulted in the loss of eyesight. A Judge can find a doctor guilty only when it is proved that he has fallen short of a standard of reasonable medical care. The fact and circumstances of the case before us show that the Appellant has attended to the patient with due care, skill, and diligence. Simply because the patient's eyesight was not restored satisfactorily, this account alone is not grounds for holding the doctor guilty of negligence and deficient in his duty. It is settled law that it is for the Complainant to prove the negligence or deficiency in service by adducing expert evidence or opinion and this fact is to be proved beyond all reasonable doubt. Mere allegation of negligence will be of no help to the Complainant.[7]

The following cases of alleged medical negligence provide an insight into how the final decision is reached by the judicial bodies. “All medical negligence cases concern various questions of fact, when we say burden of proving negligence lies on the Complainant, it means he has the task of convincing the court that his version of the facts is the correct one”. No expert opinion has been produced by the Complainant to contradict the report of the Board of Doctors. The appeal of the Complainant was dismissed with costs as “No expert opinion has been produced by him.”[8] In a case of an improper union of the patella, no expert has been produced by the Complainant to prove negligence of the opposite party. Thus, it cannot be said with exactness that treatment of the Complainant by the opposite party was against the norms prescribed under the medical jurisprudence or that the opposite party in any way was negligent or deficient in the performance of his duties.[8]

“Allegation of medical negligence is a serious issue and it is for the person who sets up the case to prove negligence based on material on record or by way of evidence”. The complaint of medical negligence was dismissed because the applicant failed to establish and prove any instance of medical negligence.[9] “Merely because the operation did not succeed, the doctor cannot be said to be negligent” and the appeal of the doctor was allowed.[10] “A mere allegation will not make a case of negligence unless it is proved by reliable evidence and is supported by expert evidence” and the appeal was dismissed.[4] “The commission cannot constitute itself into an expert body and contradict the statement of the doctor unless there is something contrary on the record by way of an expert opinion or there is any medical treatise on which reliance could be based” and the Revision petition of the doctor was allowed.[1] In another case, an X-ray report indicated a small opacity that similar to an opaque shadow that becomes visible for many causes other than a calculus. It could not be assumed that still stone existed in the right kidney that had not been operated upon. Under the circumstances, we do not think that any case of negligence has been made by the Complainant. This petition is, therefore, allowed.[11]

### The Need for Expert Evidence in Medical Negligence Cases

The Commission cannot constitute itself into an expert body and contradict the statement of the doctor unless there is something contrary on the record by way of an expert opinion or there is any medical treatise on which reliance could be based.[12] In this case there was a false allegation of urinary stone not being removed as shown by a shadow in the xray “The burden of proving the negligent act or wrong diagnosis was on the Complainant” and the appeal was dismissed in another case of alleged medical negligence as no expert evidence was produced.[13] The case discussed below is not a case of apparent negligence on the part of the surgeon in conducting the operation, but about the quality of the plate used for fixing the bone. In the present case, the Complainant has not produced any expert witnesses to prove that there was any fault in the performance of the operations. Fixation of the bones by using plates is one of the recognized modes of treatment in the case of fracture of the bones. If the opposite party has adopted the aforesaid method, though subsequently the plate broke, negligence cannot be attributed to the doctor. This is not a case where the wounds of the operation were infected or any other complication arose. Breaking of the plate approximately 6 months after it was placed cannot be attributed towards a negligent act of the doctor in performing the operation. The District Forum rightly held that the Complainant had failed to prove his case.[14] There is nothing on the record to suggest that there has been any negligence and/or deficiency in service on the part of the Appellant except the oral submission of the Respondent/Complainant. In such cases, before coming to a positive finding, there must be expert evidence on record as has been held both by the National Commission as well as the Apex Court.[15] “As per the settled law, the onus to prove that there was negligence” deficiency in service on the part of the opposite parties, while diagnosing and treating the Complainant, lay heavily on the Complainant. In the given facts, the Complainant has failed to discharge the onus that was on him. The complaint was dismissed as the Complainant failed to discharge the onus to prove negligence or deficiency in service.[16]

In medical negligence cases, it is for the patient to establish his case against the medical professional and not for the medical professional to prove that he acted with sufficient care and skill. Refer to the decision of the Madhya Pradesh High Court in the case of Smt. Sudha Gupta and Ors. *vs*. State of M.P. and Ors., 1999 (2) MPLJ 259. The National commission has also taken the same view observing that a mishap during operation cannot be said to be deficiency or negligence in medical services. Negligence has to be established and cannot be presumed. Refer to the decision of the National Commission in the case of Kanhiya Kumar Singh *vs*. Park Medicare and Research Centre, III (1999) CPJ 9 (NC) – (2000) NCJ (NC) 12. A similar view has been taken by the MRTP Commission in the case of P.K. Pandey *vs*. Sufai Nursing Home, I (1999) CPJ 65 (MRTP) – 2000 NCJ (MRTP) 268. Followed by this, refer to the Commission in Vaqar Mohammed Khan and Anr. *vs*. Dr. S. K. Tandon, II (2000) CPJ 169.[17] Both the lower Fora have held that there is no evidence brought on record by the Complainant to show that there was any negligence by the Respondent while implanting the lens in the eye of the Complainant resulting in a persistent problem in the left eye.[18]

The Complainant does not examine any expert on the subject to establish his allegation of negligence on the part of the doctor. Unfortunate though the incident is, the Complainant needs to establish negligence on the part of the doctor to succeed in a case like this. We may observe that there is hardly any cogent material to substantiate the allegation contained in the petition of Complainant. Under the circumstances, we cannot but hold that the Complainant has failed to prove the allegations against the opposite parties.[19] As held by the National Commission in Sethuraman Subramaniam Iyer *vs*. Triveni Nursing Home and anr., 1998 CTJ7, in the absence of such evidence regarding the cause of death and absence of any expert medical evidence, the Complainants have failed to prove negligence on the part of the opposite parties.[20]

In order to decide whether negligence is established in any particular case, the alleged act, omission, or course of conduct that is the subject of the complaint must be judged not by ideal standards nor in the abstract but against the background of the circumstances in which the treatment in question was given. The true test for establishing negligence on the part of a doctor is as to whether he has been proven guilty of such failure as no doctor with ordinary skills would be guilty of if acting with reasonable care. Merely because a medical procedure fails, it cannot be stated that the medical practitioner is guilty of negligence unless it is proved that the medical practitioner did not act with sufficient care and skill and the burden of proving this rests upon the person who asserts it. The duty of a medical practitioner arises from the fact that he does something to a human being that is likely to cause physical damage unless it is not done with proper care and skill. There is no question of warranty, undertaking, or profession of a skill. The standard of care and skill to satisfy the duty in tort is that of the ordinary competent medical practitioner exercising an ordinary degree of professional skill. As per the law, a defendant charged with negligence can clear himself if he shows that he acted in accordance with the general and approved practice. It is not required in the discharge of his duty of care that he should use the highest degree of skill, since this may never be acquired. Even a deviation from normal professional practice is not necessary in all cases evident of negligence.[21]

## RECENT SUPREME COURT'S JUDGMENT

The recent judgment pronounced in Martin F. D'Souza V. Mohd. Ishfaq[22] by the Hon'ble Supreme Court of India quite explicitly addresses the concerns of medical professionals regarding the adjudicatory process that is to be adopted by Courts and Forums in cases of alleged medical negligence filed against Doctors.

In March 1991, the Respondent who was suffering from chronic renal failure was referred by the Director of Health Services to the Nanavati Hospital in Mumbai for the purpose of a kidney transplant. At that stage, the Respondent was undergoing hemodialysis twice a week and was awaiting a suitable kidney donor. On May 20, 1991, the Respondent approached the Appellant doctor with a high fever, but he refused hospitalization despite the advice of the Appellant. On May 29, 1991 the Respondent who still had a high fever finally agreed to get admitted into the hospital due to his serious condition. On June 3, 1991, the reports of the urine culture and sensitivity showed a severe urinary tract infection due to Klebsiella species (1 lac/ml) sensitive only to Amikacin and Methenamine Mandelate. Methnamine Mandelate cannot be used in patients suffering from renal failure. Since the urinary infection was sensitive only to Amikacin, an injection of Amikacin was administered to the Respondent for 3 days (from June 5, 1991 to June 7, 1991). Upon treatment, the temperature of the Respondent rapidly subsided. On June 11, 1991, the Respondent who presented to the hemodialysis unit complained to the Appellant that he had slight tinnitus (ringing in the ear). The Appellant has alleged that he immediately told the Respondent to stop taking the Amikacin and Augmentin and scored out the treatment on the discharge card. However, despite express instructions from the Appellant, the Respondent continued taking Amikacin until June 17, 1991. Thereafter, the Respondent was not under the treatment of the Appellant. On June 14, 1991, June 18, 1991, and June 20, 1991 the Respondent received hemodialysis at Nanavati Hospital and allegedly did not complain of deafness during this period. On June 25, 1991, the Respondent, on his own accord, was admitted to Prince Aly Khan Hospital. The Complainant allegedly did not complain of deafness during this period and conversed with doctors normally, as is proved from their evidence. On July 30, 1991, the Respondent was operated upon for a transplant and on August 13, 1991, the Respondent was discharged from Prince Aly Khan Hospital after his transplant. The Respondent returned to Delhi on August 14, 991 after his discharge.

On July 7, 1992, the Respondent filed a complaint before the National Consumer Disputes Redressal Commission, New Delhi claiming compensation of an amount of Rs.12,00,000/- as his hearing had been affected. The Appellant filed his reply stating, inter alia, that there was no material brought on record by the Respondent to show any co-relationship between the drugs prescribed and the state of his health. The National Consumer Disputes Redressal Commission passed an order on October 6, 1993 directing the nomination of an expert from the All India Institute of Medical Sciences, New Delhi (AIIMS) to examine the complaint and give an unbiased and neutral opinion. AIIMS nominated Dr. P. Ghosh who was of the opinion that the drug Amikacin was administered by the Appellant as a life-saving measure and was rightly used. It is submitted by the Appellant that the said report further makes it clear that there has been no negligence on the part of the Appellant. However, the National Commission has come to the conclusion that the Doctor was negligent.

### Supreme Court's Appreciation with Regard to Medical Negligence Liability

According to the Supreme Court, cases both civil and criminal as well as in Consumer Fora, are often filed against medical practitioners and hospitals complaining of medical negligence against doctors, hospitals, or nursing homes, hence the latter would naturally like to know about their liability. The general principles on this subject have been lucidly and elaborately explained in the three Judge Bench decisions of this Court in Jacob Mathew *vs*. State of Punjab and Anr. (2005) 6 SCC 1. However, difficulties arise in the application of those general principles to specific cases. For instance, in paragraph 41 of the decision, it was observed that: “The practitioner must bring to his task a reasonable degree of skill and knowledge and must exercise a reasonable degree of care. Neither the very highest nor a very low degree of care and competence is what the law requires.” Now what is reasonable and what is unreasonable is a matter on which even experts may disagree. Also, they may disagree on what is a high level of care and what is a low level of care. To give another example, in paragraphs 12 to 16 of Jacob Mathew's case (Supra), it has been stated that simple negligence may result only in civil liability, but gross negligence or recklessness may result in criminal liability as well. For civil liability only, damages can be imposed by the Court but for criminal liability the Doctor can also be sent to jail (apart from damages that may be imposed on him in a civil suit or by the Consumer Fora). However, what is simple negligence and what is gross negligence may be a matter of dispute even among experts.

The law, like medicine, is an inexact science. One cannot predict with certainty an outcome in many cases. It depends on the particular facts and circumstances of the case, and also the personal notions of the Judge who is hearing the case. However, the broad and general legal principles relating to medical negligence need to be understood. Before dealing with these principles two things have to be kept in mind:

1. Judges are not experts in medical science, rather they are laymen. This itself often makes it somewhat difficult for them to decide cases relating to medical negligence. Moreover, Judges usually have to rely on the testimonies of other doctors, which may not be objective in all cases. Since like in all professions and services, doctors too sometimes have a tendency to support their own colleagues who are charged with medical negligence. The testimony may also be difficult to understand for a Judge, particularly in complicated medical matters and
2. a balance has to be struck in such cases. While doctors who cause death or agony due to medical negligence should certainly be penalized, it must also be remembered that like all professionals doctors too can make errors of judgment but if they are punished for this no doctor can practice his vocation with equanimity. Indiscriminate proceedings and decisions against doctors are counter productive and are no good for society. They inhibit the free exercise of judgment by a professional in a particular situation.

### The reasoning and decision

In the words of the Supreme Court, the facts of the case reveal that the Respondent was suffering from chronic renal failure and was undergoing hemodialysis twice a week as treatment. He was suffering from a high fever but he refused to get admitted into the hospital despite the advice of the Appellant. The Respondent was also suffering from a severe urinary tract infection that could only be treated by Amikacin or Methenamine Mandelate. Since Methenamine Mandelate cannot be used for patients suffering from renal failure, an injection of Amikacin was administered. A perusal of the complaint filed by the Respondent before the National Commission shows that his main allegation was that he suffered from a hearing impairment due to the negligence of the Appellant who allegedly prescribed an overdose of Amikacin injections with no regard for the critical condition of the Respondent who did not warrant such heavy dosage.

The case of the Appellant, however, is that the Complainant was referred to the Appellant by Dr. F.P. Soonawalla, the renowned Urologist of Bombay. Dr. Soonawalla is an eminent doctor of international repute and he would not have ordinarily referred a patient to an incompetent doctor. This is one factor that goes in favor of the Appellant, though of course it is not conclusive. After examining the Complainant, the Appellant found that the Complainant was a patient of chronic renal failure due to bilateral polycystic kidneys and the Appellant advised hemodialysis twice a week as an out-patient. The Complainant was also investigated to find a suitable kidney donor. The Appellant has alleged in his written statement filed before the National Commission that the Complainant was in a hurry to have a quick kidney transplant and he was very obstinate, stubborn, and short-tempered.

The Appellant was of the view that the Respondent's infection could only be treated by an injection of Amikacin, as Methenamine Mandelate could not be used due to his chronic renal failure. The Respondent's report also established his resistance to all other antibiotics. In our opinion, it is clear that the Respondent already had renal failure before the injection of Amikacin. Amikacin was administered after a test dosage only from June 5, 1991 and at this stage he did not complain of any side effects and his temperature subsided rapidly. On June 11, 1991, the Respondent complained to the Appellant of slight tinnitus or ringing in the ear. The Appellant immediately reviewed the treatment on the discharge card in possession of the Respondent and also asked his attendant i.e., his wife, to stop the injection of Amikacin and Cap. Augmantine verbally and also marked an X on the discharge card in his own handwriting on June 11, 1991 i.e., 3 days after discharge. Hence, as per the direction of the Appellant, the Respondent should have stopped receiving injections of Amikacin after June 10, 1991, but on his own he kept taking Amikacin injections. On perusal of the copies of the papers from the Cash Memo supplied by the Respondent as per annexure 4, it is in our opinion evident that the Respondent continued to take the medicine against the advice of the Appellant, and had unilaterally been getting injected as late as June 17, 1991, i.e., 7 days after he had been instructed verbally and in writing in the presence of his attendant i.e., his wife and staff members of the hospital to stop injections of Amikacin/Cap. Augmantine because of tinnitus as early as June 11, 1991. From the above facts, it is evident that the Appellant was not to blame in any way and it was the non cooperative attitude of the Respondent and his continuing with the Amikacin injections even after June 11, 1991 that was the cause of his ailment, i.e., the impairment of his hearing. A patient who does not listen to his doctor's advice often has to face adverse consequences. It is evident from the fact that the Respondent was already seriously ill before he met the Appellant. There is nothing to show from the evidence that the Appellant was in any way negligent, rather it appears that the Appellant did his best to give good treatment to the Respondent to save his life but the Respondent himself did not cooperate.

Several doctors have been examined by the National Commission and we have read their evidence, which is on record. Apart from that, there is also the opinion of Prof. P. Ghosh of the All India Institute of Medical Sciences who had been nominated by AIIMS as requested by the Commission, which is also on record. The opinion of Dr. Ghosh was that there were many factors in the case of renal diseases that cause hearing loss and it is impossible to foretell the sensitivity of a patient to a drug, thereby making it difficult to assess the contributions towards toxicity by the other factors involved. He has also opined that the Amikacin dose of 500 mg twice a day for 14 days prescribed by the doctor was a life-saving measure and the Appellant did not have any option but to take this step. Life is more important than saving the function of the ear. Prof Ghosh was of the view that antibiotics were rightly given on the report of the sensitivity test that showed the organisms were sensitive to Amikacin. Hence, the antibiotic was not blindly used on speculation or as a clinical experiment. In view of the opinion of Prof Ghosh, who is an expert of the All India Institute of Medical Sciences, we are clearly of the view that the Appellant was not guilty of medical negligence but rather wanted to save the life of the Respondent. The Appellant was faced with a situation where not only was there kidney failure of the patient, but also urinary tract infection and blood infection. In this grave situation, which threatened the life of the patient, the Appellant had to take drastic steps. Even if he prescribed Amikacin for a longer period than is normally done, he obviously did it to save the life of the Respondent. We have also seen the evidence from other doctors as well as the affidavits filed before the National Commission. No doubt some of the doctors who have deposed in this case have given different opinions, but in cases relating to allegations of medical negligence, this Court has to exercise great caution. From these depositions and affidavits it cannot be said that the Appellant was negligent. In fact, most of the doctors who have deposed or given their affidavits before the Commission have stated that the Appellant was not negligent.

We see no reason to disbelieve the above allegations of the Appellant that on June 11, 1991 he had asked the Respondent to stop taking Amikacin injections, and in fact this version is corroborated by the testimony of the Senior Sister Mukta Kolekar. Hence, it was the Respondent himself who is to blame for having continued Amikacin after June 11, 1991 against the advice of the Appellant. Moreover, in the statement of Dr. Ghosh before the National Consumer Dispute Redressal Commission it has been stated that it is by no means established that Amikacin alone can cause deafness. Dr. Ghosh stated that there are 8 factors that can cause loss of hearing. Moreover, there are conflicting versions about the deafness of the Respondent. While the Respondent stated that he became deaf in June 1991, most of the Doctors who filed affidavits before the Commission have stated that they freely conversed with him in several meetings much after 21st June and in fact up to the middle of August 1991.

The National Commission had sought the assistance of AIIMS to give a report about the allegations of medical negligence against the Appellant. AIIMS had appointed Dr. Ghosh to investigate the case and submit a report and Dr. Ghosh submitted a report in favor of the Appellant. Surprisingly, the Commission has not placed much reliance on the report of Dr. Ghosh, although he is an outstanding ENT specialist of international repute. We have carefully perused the judgment of the National Commission and we regret that we are unable to concur with the views expressed therein. The Commission, which consists of laymen in the field of medicine, has sought to substitute its own views over that of medical experts, and has practically acted as super-specialists in medicine. Moreover, it has practically brushed aside the evidence of Dr. Ghosh, whose opinion was sought on its own direction, as well as the affidavits of several other doctors (referred to above) who have stated that the Appellant acted correctly in the situation he was faced. The Commission should have realized that different doctors have different approaches, for instance, some have more radical approaches while some have more conservative approaches. All doctors cannot be fit into a straight-jacketed formula and cannot be penalized for departing from that formula.

While this Court has no sympathy for doctors who are negligent, it must also be said that frivolous complaints against doctors have increased by leaps and bounds in our country particularly after the medical profession was placed within the purview of the Consumer Protection Act. To give an example, earlier when a patient who had a symptom of having a heart attack would come to a doctor, the doctor would immediately inject him with Morphia or Pethidine injection before sending him to the Cardiac Care Unit (CCU) because in cases of heart attack time is the essence of the matter. However, in some cases the patient died before he reached the hospital. After the medical profession was brought under the Consumer Protection Act vide Indian Medical Association *vs*. V.P. Shantha 1995 (6) SCC 651 doctors who administer the Morphia or Pethidine injection are often blamed and cases of medical negligence are filed against them. The result is that many doctors have stopped giving (even as family physicians) Morphia or Pethidine injections even in emergencies despite the fact that from the symptoms the doctor honestly thought the patient was having a heart attack. This was out of fear that if the patient died the doctor would have to face legal proceedings. Similarly, in cases of head injuries (which are very common in road side accidents in Delhi and other cities) earlier the doctor who was first approached would started giving first aid and apply stitches to stop the bleeding. However, now what is often seen is that doctors out of fear of facing legal proceedings do not give first aid to the patient, and instead tell him to proceed to the hospital by which time the patient may develop other complications.

Hence, Courts and Consumer Fora should keep the above factors in mind when deciding cases related to medical negligence, and not take a view that would be in fact a disservice to the public. The decision of this Court in Indian Medical Association *vs*. V.P. Shantha (Supra) should not be understood to mean that doctors should be harassed merely because their treatment was unsuccessful or caused some mishap which was not necessarily due to negligence. In fact, in the aforementioned decision, it has been observed that (vide para 22): “In the matter of professional liability professions differ from other occupations for the reason that professions operate in spheres where success cannot be achieved in every case and very often success or failure depends upon factors beyond the professional man's control.”

It may be mentioned that the All India Institute of Sciences has been doing outstanding research in Stem Cell Therapy for the last 8 years for treating patients suffering from paralysis, terminal cardiac condition, parkinsonism, etc., though not yet with very notable success. This does not mean that the work of Stem Cell Therapy should stop, otherwise science cannot progress.

We, therefore, direct that whenever a complaint is received against a doctor or hospital by the Consumer Fora (whether District, State, or National) or by the Criminal Court, before issuing notice to the doctor or hospital against whom the complaint was made the Consumer Forum or Criminal Court should first refer the matter to a competent doctor or committee of doctors specialized in the field relating to which the medical negligence is attributed. Only after that doctor or committee reports that there is a prima facie case of medical negligence should a notice be issued to the concerned doctor/hospital. This is necessary to avoid harassment to doctors who may not be ultimately found to be negligent. We further warn the police officials not to arrest or harass doctors unless the facts clearly come within the parameters laid down in Jacob Mathew's case (supra), otherwise the policemen will themselves have to face legal action.

In the present case, the Appellant was faced with an extremely serious situation. Had the Appellant been only suffering from renal failure, it is possible that a view could be taken that the dose prescribed for the Appellant was excessive. However, the Respondent was not only suffering from renal failure but he was also suffering from urinary tract infection and blood infection i.e., septicemia, which is blood poisoning caused by bacteria or a toxin. He also had extremely high urea. In this extremely serious situation, the Appellant naturally had to take a drastic measure to attempt to save the life of the Respondent. The situation was aggravated by the non cooperation of the Respondent who seems to be of an assertive nature as deposed by the witnesses. Extraordinary situations require extraordinary remedies. Even assuming that such a high dose of Amikacin would ordinarily lead to hearing impairment, the Appellant was faced with a situation between the devil and the deep sea. If he chose to save the life of the patient rather than his hearing surely he cannot be faulted. The allegation against the Appellant is that he gave an overdose of the antibiotic. In this connection it may be mentioned that antibiotics are usually given for a minimum of 5 days, but there is no upper limit to the number of days for which they should continue and it all depends on the condition of the patient. Giving a lower dose of the antibiotic may create other complications because it can cause resistance in the bacteria to the drug, and then it will be more difficult to treat. With regard to the impairment of hearing of the Respondent, it may be mentioned that there is no known antibiotic drug without side effects. Hence, merely because there was impairment in the hearing of the Respondent that does not mean that the Appellant was negligent. The Appellant was desperately trying to save the life of the Respondent, which he succeeded in doing. Life is surely more important than side effects.

For example many anti-tubercular drugs (e.g., Streptomycin) can cause impairment of hearing. Does this mean that TB patients should be allowed to die and not be given the anti-tubercular drug because it impairs hearing? Surely the answer will be negative.

The courts and Consumer Fora are not experts in medical science and must not substitute their own views over that of specialists. It is true that the medical profession has to an extent become commercialized and there are many doctors who depart from their Hippocratic oath for their selfish ends of making money. However, the entire medical fraternity cannot be blamed or branded as lacking in integrity or competence just because of some bad apples. It must be remembered that sometimes despite their best efforts the treatment of a doctor fails. For instance, sometimes despite the best effort of a surgeon, the patient dies. That does not mean that the doctor or the surgeon must be held to be guilty of medical negligence, unless there is some strong evidence to suggest that he is. On the facts of this particular case, we are of the opinion that the Appellant was not guilty of medical negligence.

## CONCLUSION

The Hon'ble Mr. Justice Markendeya Katju has done yeoman service for society by rendering this judgment. On one hand, it sets at rest the speculative nature of our judicial adjudication of medical negligence liability and on the other, it abundantly clarifies that unless there is prima facie evidence indicating medical negligence, notice either to a doctor or hospital cannot be issued. At the same time, the core essence of the judgment makes it very clear that there cannot be an assumption that doctors cannot be negligent while rendering care and treatment. I think this timely intervention should be disseminated at a popular level so that the mandated Supreme Court's prescription will be observed more in practice than in breach.
